# Transfer of Metal(loid)s from Soil to Leaves and Trunk Xylem Sap of Medicinal Plants and Possible Health Risk Assessment

**DOI:** 10.3390/ijerph19020660

**Published:** 2022-01-07

**Authors:** Ana C. Gomes Rosa, Elaine S. de Pádua Melo, Ademir S. A. Junior, Jacqueline M. S. Gondim, Alexsandro G. de Sousa, Claudia A. L. Cardoso, Lucilene F. Viana, Alexandra M. A. Carvalho, David J. Machate, Valter Aragão do Nascimento

**Affiliations:** 1 Group of Spectroscopy and Bioinformatics Applied Biodiversity and Health (GEBABS), Graduate Program in Health and Development in the Central-West Region of Brazil, Federal University of Mato Grosso do Sul, Campo Grande 79079-900, Brazil; anacarlagomesrosa76@gmail.com (A.C.G.R.); elaine.melo@ufms.br (E.S.d.P.M.); junioralvesms@hotmail.com (A.S.A.J.); jackiegondim@gmail.com (J.M.S.G.); 2 Departamento de Ciências Exatas e Naturais, Campus de Itapetinga, Universidade Estadual do Sudoeste da Bahia, BR 415, KM 03, S/Nº, Primavera, Itapetinga 45700-000, Brazil; gamasousa@yahoo.com.br; 3 Centro de Estudos em Recursos Naturais, UEMS, Dourados 79804-970, Brazil; claudia@uems.br; 4 Programa de Pos-Graduacao EM Ciencias e Tecnologia Ambiental (CTA), Faculdade de Ciencias Exatas e Tecnologia—FACET, Universidade Federal da Grande Dourados (UFGD) Cidade Universitaria, Rodovia Dourados Itahum, Km 12, Caixa Postal 364, Dourados 79804-970, Brazil; lucilenefinoto@hotmail.com; 5 Graduate Program in Health and Development in the Central-West Region of Brazil, Federal University of Mato Grosso do Sul, Campo Grande 79079-900, Brazil; alexandra.carvalho@ufms.br; 6 Graduate Program in Sciences of Materials, Federal University of Mato Grosso do Sul, Campo Grande 79079-900, Brazil; machatedavidjohanemachate@yahoo.com.br

**Keywords:** vehicular traffic, metal(loid)s, landfill, highway, trunk xylem sap, medicinal plants, health effects, *Dipteryx alata* Vog

## Abstract

The objective of the present study was to investigate metal(loid)s in soils, in the trunk xylem sap and in the leaves of the *Dipteryx alata* plant located near the highway with high vehicle traffic in agricultural regions and near landfills, and to assess the transfer of metal(loid)s from soil to plant and possible health risk assessment. Trunk xylem sap, leaves and soil samples were collected at three sites near the highway. The analysis of trace elements was carried out using inductively coupled plasma optical emission spectroscopy (ICP OES). In the three soil sampling sites far from the highway edge, 15 elements were quantified. The concentrations of elements in the soil presented in greater proportions in the distance of 5 m in relation to 20 and 35 m. The metal(loid)s content in the study soil was higher than in other countries. The concentrations of Al, Cu, Fe, Mg, Mn, P, Se and Zn in the xylem sap were much higher than the leaves. The values of transfer factor of P, Mg and Mn from soil to the xylem sap and transfer factor of P from soil to leaf were greater than 1, indicating that the specie have a significant phytoremediation and phytoextraction potential. This plant has a tendency to accumulate As, Cd and Cr in its leaf tissues. The chronic hazard index (HI) values recorded in this study were above 1 for adults and adolescents. It is concluded that the soil, the trunk xylem sap and leaves of this plant are contaminated by heavy metals. Ingestion of the trunk xylem sap of this plant can cause toxicity in humans if ingested in large quantities and in the long term; therefore, its consumption should be avoided.

## 1. Introduction

In the last decades, vehicular traffic has been one of the main causes of air and soil pollution in urban and rural areas. According to epidemiological studies, there is an association between exposure to soil pollutants and adverse health effects [1,2,3]. Many cars and trucks run on highways and county roads, spreading pollution across several countries. Soil contamination by heavy metals, metalloids, ammonia, nitrate, petroleum hydrocarbons, naphthalene, herbicide and pesticides and other contaminants in excessive amounts is one of the great environmental problems of the soil [4]. Thus, pollution harms the soils and plants and enters our food [5]. According to Food and Agriculture Organization of the United Nations (FAO), it is estimated that 95 percent of food is directly or indirectly produced on soils [6]. Although several researchers have developed methodologies to reduce vehicle pollution [7], there remains a need for further studies to gain an understanding of all contaminants, and their environmental and human health impacts, as well as in plants and animals.

According to the European Environment Agency (EEA), directives and regulations, as well as and new technologies, have contributed to the reduction of heavy metals in the environment. In 2017, EEA-33 emissions of Pb had declined to less than one tenth of total 1990 emission levels due to reductions made by countries in emissions from the road transport sector, that is, unleaded petrol [8]. All the success of this decline in Pb decrease was brought about by fiscal and regulatory measures. In addition, the emissions of Cd declined by approximately 35% from 1990 to 2017 because of news abatement technologies for wastewater treatment and incinerators, modern smelting facilities and metal refining, directives and regulations that mandate reductions, and limits on heavy metal emissions [9]. In all EEA-33 countries, emissions of mercury (Hg) decreased by 30% from 1990. This decrease of Hg is attributed to improvements in emission controls on Hg cells and their replacement by diaphragm or membrane cells, and fuel switching from coal to gas.

Studies have shown that fruits collected near roads accumulate heavy metals and metalloids [10]; however, there are few studies involving medicinal plants collected near highway with high vehicle traffic. Thus, risk assessment [11] and transfer factors of metal(loid)s between two consecutives levels of the chain as soil and medicinal plants are essential to carry out environmental monitoring and ensure consumer safety due to plant ingestion medicinal [12,13].

In Brazil cerrado biome, there are several species of plants, *Dipteryx alata* (*D. alata*) Vog (Leguminosae), popularly known as “cambaru or baru”, that stand out as a common species in this biome [14]. The pulp and seeds of *D. alata* are used in the manufacture of ice cream, cream, liqueur [15], and the pulp extract serves to treat urinary tract infections [16]. In addition, studies in vitro shows that the neurotoxic and myotoxic actions of *Bothrops jararacuçu* venom were decreased by the methanolic extract from *D. alata* bark [17]. According to traditional knowledge in some regions of Brazil, the trunk xylem sap of the *D. alata* serves as a tonic. Baru seed has high levels of lipids, proteins and minerals such as potassium, phosphorus and magnesium. Conversely, seed oil has a high degree of unsaturation, α-tocopherol, fatty acids, oleic and linoleic acids [18].

To date, there are only studies that have quantified element chemicals in the leaves [19] and almonds of *D. alata* collected near rural areas [20,21]. However, no studies were carried out to evaluate the chemical elements in soils, trunk xylem sap and *D. alata* leaves collected near highway with heavy vehicle traffic in agricultural regions and near landfills. Heavy metal and metalloids contamination of soil may pose risks and hazards to humans and the ecosystem. At this point, fuels, lubricating oils, fertilizers, tires and vehicle brake discs are the main source of chemical elements as iron (Fe), copper (Cu), zinc (Zn), scandium (Sn), aluminum (Al), silicon (Si), zirconium (Zr), titanium (Ti), antimony (Sb), chromium (Cr), molybdenum (Mo), manganese (Mn), vanadium (V), Nickel (Ni), Bismuth (Bi), phosphor (P), lead (Pb), cobalt (Co), arsenic (As) and cadmium (Cd) [22,23,24,25,26].

Therefore, the main objective of this study was: (i) to quantify Al, As, Cd, Co, Cr, Cu, Fe, Mg, Mn, Mo, Ni, P, Se, V and Zn in the trunk xylem sap and leaves of the *D. alata* plant, which is localized to 35 m from the highway with high vehicle traffic in agricultural regions and near to landfills in Campo Grande, Brazil, (ii) to quantify the concentration of Al, As, Cd, Co, Cr, Cu, Fe, Mg, Mn, Mo, Ni, P, Se, Zn and Pb in soil collected at three sampling sites transections perpendicular to the highway at distances of 5, 20 and 35 m, (iii) to calculated the transfer factor from soil to trunk xylem sap and transfer factor from soil to leaves of the plant, (iv) and also to evaluate whether the use of the trunk xylem sap is safe to consumer according to health risk assessment.

## 2. Materials and Methods

### 2.1. Schematic Drawing of the Study

**Figure 1:**
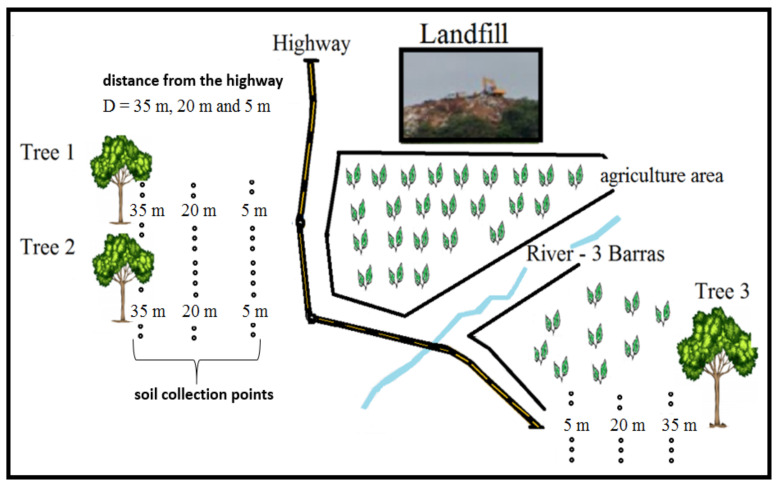
Schematic drawing of the sampling location—trees 1, 2 and 3 (plant samples and distance from the highway D = 5, 20 and 35 m), the three parallel lines (soil sample) from both sides of the MS-40 highway, and surroundings (river, landfill and agricultural areas).

Three trees located close to a highway with high vehicular traffic, as well as close to a landfill and an area of intense agriculture, were selected for this study (Figure 1). The soil and plant samples were collected by sampling transections perpendicular to the highway at distances of D1 = 5 m, D2 = 20 m and D3 = 35 m from the highway edge (see Figure 1). The distance between trees 1 and 2 was 26.4 m, and between trees 1 and 3 was 864 m and between trees 2 and 3 was 837.50 m. The distance between the landfill and the highway was approximately 30 m. The distance between the landfill and trees 1 and 2 was 600 m, and between the landfill and tree 3 was 1450 m.

This study analyzed leaves and xylem sap from the trunk of three *D. alata* trees and samplings from the surrounding soil. The project was registered in the National Genetic Resource Management System and Associated Traditional Knowledge (SisGen, # A7716EC). The trees are located on the banks of a highway (MS-040), between the city of Campo Grande and Rio Pardo, Mato Grosso do Sul, Brazil (coordinates, latitude -20570953; longitude -54551781). The road has a flow of approximately 4200 vehicles per day. Nearby, at around 30 m, on the right side of the road is a landfill and some agricultural areas. On the left side of the road are trees 1 and 2, 26.4 m apart from each other, and across the road is tree 3, at 864 m from tree 1 and 837.5 m from tree 2. Soil samples were collected on both sides of the road, in three parallel lines distant 5 m, 20 m and 35 m from both sides of the highway edge in the direction of the trees. A schematic drawing of the area is shown in Figure 1.

### 2.2. Sample Collection

**Figure 2:**
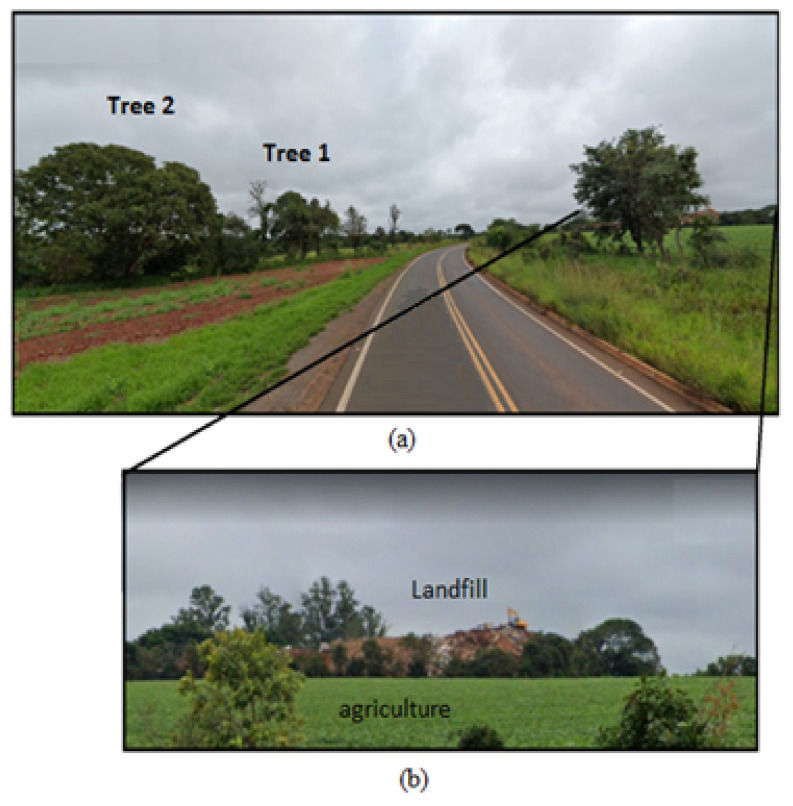
(**a**) Location of trees 1 and 2 near highway edge. (**b**) Landfill near the highway and bean plantation (agriculture).

Tree and soil samplings were taken in June 2021. Figure 2 shows the position of trees near the highway, landfill and agricultural region (bean plantation). The content analysis of the trees consisted of samples of leaves and trunk xylem sap. One hundred grams of leaves were collected from each tree and stored in sterile sample bags. A composite sample was formed from the leaf samples collected at each sampling site (see Figure 1, D3). We collected samples of trunk xylem sap of each tree. First, we drilled a hole in the trunk 1.4 m off the ground using a 0.5 m long (15 mm diameter) steel drill. We then inserted a hose into the hole to collect 200 mL of xylem sap per tree. These samples were mixed to obtain a representative sample.

A total of ninety medium textured soil samples (type 2) were collected between the trees and the highway. We collected soil samples in three parallel lines at 5, 20, and 35 m on the left side of the road toward trees 1 and 2 and likewise on the right side of the road toward tree 3 (Figure 1), which resulted six lines.

Using a stainless-steel shovel, we collected samples of 100 g of soil, at 20 cm from the surface, at 15 sites spaced 1.5 m apart for each of the six rows. We blended the samples from each line equidistant from the road toward the trees on both sides to form a representative soil sample 5, 20, and 35 m from the road.

### 2.3. Sample Preparation and Digestion

The samples (300 mg) of *D. alata* leaves were placed in an oven and subjected to a drying process at 40 °C for 10 h until reaching a constant weight. The dried samples were crushed separately with a portable stainless steel electric grinder to obtain a very fine powder (Termomix, Brazil) and then sieved (stainless steel sieve, 200 μm granulometry). Approximately 0.25 g of the leave sample powder was placed in a Teflon DAP60^®^ vessels and added 3.0 mL of HNO_3_ (65%, Merck, Darmstadt, Germany), 1.0 mL of high-purity water (18 MΩ cm, Milli-Q, Millipore, Bedford, MA, USA) and 2.0 mL of H_2_O_2_ (35%, Merck, Darmstadt, Germany). The digestion procedure using microwave digestion system (Speedwavefour, Berghof, Germany) was performed according to Ref. [27]. However, there is another method where elements are extracted from the leaf surfaces using dilute nitric acid in the sample-collection bottle [28].

About 5 mL of trunk xylem sap samples were accurately weight into Teflon digestion vessel. Next, 1 mL of HNO_3_ (65% Merck, Darmstadt, Germany) and 1.5 mL de H_2_O_2_ (35%, Merck Millipore, Darmstadt, Germany) were added. Trunk xylem sap samples in solution were prepared using the Vortex shakers during 5 min and dilute up to 30 mL with ultrapure water.

The collected soil samples were air-dried until constant weight was obtained, then ground and sieved in a 2 mm sieve as per Ref. [29]. An amount of 0.500 g of soil samples was weighted directly into Teflon DAP60^®^ vessels (Berghof Products + Instruments GmbH, Eningen, Germany) and 9 mL of HCl (35%, Merck, Darmstadt, Germany) and 3 mL of H_2_O_2_ (65%, Merck, Darmstadt, Germany) was added and leaving at rest for pre-digestion for 18 h with the DAP60^®^ vessel loosely capped to allow gases to escape. After predigestion, the samples were digested according to the recommendations in USEPA method 3051A guidelines [30]. After cooling, the samples were filtered, transferred into 25 mL volumetric flasks and made up to the mark with ultrapure water. All digestion soils, trunk xylem sap and leaf samples were analyzed in triplicate.

### 2.4. Sample Analysis

The chemical elements were quantified by ICP OES with an axial plasma (iCAP 6300 Duo, Thermo Fisher Scientific, Bremen, Germany). ICP OES operating condition areas following power = 1250 W; sample flow rate = 0.35 L·mn^−1^; plasma gas flow rate = 12 L·mn^−1^; integration time = 5 s; stabilization time = 20 s; pressure of nebulization = 20 psi; plasm view = axial, gas view: air. In addition, the following emission wavelengths (nm) were setup and were used by the ICP OES for analysis of each of the elements: Al 309.271 nm, Pb 220.353 nm, As 189.042 nm, Cu 324.754 nm, Fe 259.940 nm, V 309.311 nm, Mg 279.553 nm, Mn 257.610 nm, Mo 202.030 nm, Se 196.00 nm, Ni 221.647 nm, Zn 213.856 nm, P 214.914 nm, Cr 267.716 nm, Co 228.616 nm, Cd 228.802 nm.

Standard solutions were prepared by diluting a standard multiple element stock solution (SpecSol, Quinlab, Brazil) containing 1000 mg/L of each element (Al, As, Cd, Co, Cr, Cu, Fe, Mg, Mn, Mo, Ni, P, S, V, Se, and Zn). For the quantitative analysis of soils and sap, external calibration curves were built on five different concentrations in the range of 0.01–5.0 mg/L. Optimal conditions were evaluated in terms of accuracy (by recovery test) and limit of detection. The spiking solution was made from a single multielement stock solution of 1000 ppm. Thus, a recovery test was performed; the solutions were spiked with 1 ppm. The method had a recovery interval of 81–112%. The limits of detection (LOD) were calculated as 3 times the standard deviation of the mean of blank sing (SB) determinations divided by calibration curve slope (Sp), that is: 3 × SB/Sp [31]. Conversely, the limits of quantification (LOQs) were calculated as follows LOQ = 10 × SB/Sp. The range of all elements LOD was 0.02–0.3 µg/L, and the range of all elements LOQ was 0.06 to 10 µg/L. The range of the correlation coefficient (*R*^2^) was 0.9993–0.9998.

### 2.5. Transfer Factor

The transmission of metal (loid) concentrations in the soils to trunk xylem sap of the tree, or transmission of metal (loid) concentrations in the soils to leaves of the tree were calculated based on dry weight (dw), obtained through an index called the Transfer Factor (*TF*). The transfer factor (*TF*) was calculated as follows [29,32]:

(1) $$TF=\frac{C_{P}}{C_{s}}$$

where *C_P_* is the metal(loid)s concentration in xylem sap of the plant (mg·kg^−1^·dw) or concentration of metal(loid)s in leaves of the tree, and *C_s_* is the metal (loid) concentration in soil (mg·kg^−1^·dw). Here, we considered that when *TF* > 1, there is higher absorption of metal from soil by the plant and higher suitability of the plant for phytoremediation and phytoextraction [33]. However, when *TF* < 1, indicating that this plant species did not have phytoremediation potential as the known hyper accumulator plants [34].

### 2.6. Estimated Daily Intake

The health risk posed to consumers was determined by the specific dietary intake of each metal, heavy metals or metalloids. The estimated daily intake (*EDI*) of metal (loid)s from the consumption of trunk xylem sap of the tree was calculated as follows:

(2) $$EDI=\frac{C_{P}\times D}{Bw}$$

where *C_P_* is described in Equation (1), and *D* stands for the daily average consumption of sap (250 mg/day for adults and 50 mg/day for adolescents), and *Bw* represents the body weight in kg (70 kg for adults and 40 kg for adolescents). In addition, the estimated daily intake (*EDI*), which is calculated in Equation (2), was compared to the upper level of tolerable intake (UL) [35]. The UL is the highest level of daily nutrient intake likely to pose no risk of adverse health effects for almost all individuals.

### 2.7. Target Hazard Quotient

The non-carcinogenic health hazards through sap consumption were evaluated by the target hazard quotient (*HQ*) using Equation (3) [29,36].

(3) $$HQ=\frac{EF\times ED}{RfD\times T}\times \frac{C_{p}\times D}{Bw}\;$$

where *EF* is exposure frequency assumed to be 90 days/year for adults and adolescents, *ED* is exposure duration 50 years for adults (female and male) and 12 years for adolescents (female and male). *RfD* is the oral reference dose (considered to be Al 1.0, Cu 4.0 × 10^−2^, Fe 7.0 × 10^−1^, Mn 2.4 × 10^−2^, P 2.0 × 10^−5^, Se 5.0 × 10^−3^, and Zn 3.0 × 10^−1^ mg/kg-day) which is an estimation of the maximum permissible risk on human population thought daily exposure [37]; however, according to chemical update worksheet, *RfD* for magnesium is 11 mg/kg-day [38]. *T* is the average exposure time for non-carcinogenic effect (*T* = *ED* × 365 days/year) [39]. In Equation (3), the terms *Cp*, *D* and *Bw* are describe in Equation (2).

If, *HQ* < 1, it represents adverse non-carcinogenic effects of concern due the daily exposure to a certain metal(loid)s through sap consumption, while *HQ* > 1 represents that in the exposure population, chronic health risk may occur [29].

To evaluate the potential risk to human health through simultaneous exposure metal(loid)s, chronic hazard index (*HI*) is obtained as the sum of all *HQs* of each metal(loid)s [40], it was calculated using the equation below:

(4) $$HI=\sum HQ=\frac{EF\times ED}{RfD\times T}\times \frac{C_{p}\times D}{Bw}$$

If *HI* < 1, sap consumption is safe; however, when *HI* > 1, trunk xylem sap consumption may pose a health risk.

### 2.8. Statistical Analysis

The Kruskal–Wallis test, along with Dunn’s test, was used to compare the concentrations of metal(loid)s in the soil at different collection distances. The significance level was set at *p* < 0.05. Normality and homogeneity were tested by the Shapiro–Wilk test. Principal component analysis was carried out to evaluate the concentrations of metal(loid)s in the soil and the distribution between the different collection distances. Principal component analysis was realized using the “dudi.pca” command in the “ade4” package [41]. In order to visualize similarity of intensities of metal(loid)s concentrations in soil samples at different distances (5, 20 and 35 m), we used a hierarchical grouping by metal(loid)s types and distances between sampling sites. The intensity of the distances metal(loid)s concentrations was centered by the mean (zero) and standard deviation (1). Using a “heat map” command in the “gplots” package, we generated a specific color-coded grouping by distances when the intensity of the metal(loid)s concentrations reached proportions outside the normal range. Color scale represents average values, where red tones indicate above-average values for a particular metal(loid)s, while blue tones indicate below-average values. All analyses were performed using the R platform [42].

## 3. Results

### 3.1. Concentration of the Meta(loid)s in Soil, Trunk Xylem Sap and Leaves of the D. alata

The concentrations of metal(loid)s in the soil collected between distances of the sampling sites (D1, D2 and D3) and the highway edge are shown in Table 1. As can been in Table 1, the concentration of elements obtained in soil at distances of 5, 20 and 35 m from highway was compared to prevention values of Brazil Environmental Council (Conama) determined from human health-based risk analysis [43] and compared to the concentration of elements in soils with agricultural, industrial and mining activities from China and USA [44]. There are no risk values for Al, Cr, Fe, Mn, Mg, P and Se in soils established by Brazil/Conama. In addition, China has not defined values for P in soils and in the United States of America for Cd.

Regarding the concentrations of the quantified elements in the soil (As, Cd, Co, Cr, Cu, Mg, Mn, Mo, Ni, P, Se, Zn and Pb), we found a significant difference between the distances from the sampling sites (D1, D2 and D3) and the highway, between the distance of 5 m compared to 20 and 35 m (*p* < 0.05) (Table 1). For the same elements, there was no difference between the distance of 20 and 35 m (*p* > 0.05) (Table 1). For Al and Fe there were no significant differences between the three distances (*p* > 0.05) (Table 1).

Comparisons between the obtained results in our soil and values reported by Brazil/Conama, China and USA show that:

- For distances D1, D2 and D3 from highway (Table 1), the results obtained for the concentration of Al, As, Cd, Co, Fe, Mg, Mo, Ni, P and Se are considerably higher than those reported by Brazil/Conama, and other countries as China and USA;
- The obtained Cr, Mn and Pb levels in three sampling sites of soil at D1, D2 and D3, are below than those reported Brazil/Conama, China and USA;
- The obtained results for Cu and Zn levels in three sampling sites of soil at D1, D2 and D3, are below than those reported by Brazil/Conama; however, these values are highest than the values set by China and USA.

The results of the concentration of chemical elements of the trunk xylem sap and leaves of *D. alata* are summarized in Table 2. Only eight elements were quantified in the plant xylem sap in the following descending order: Mg > P > Mn > Al > Se > Fe > Zn > Cu; however, their results showed that the concentration of As, Cd, Co, Cr, Mo, Ni, and V in trunk xylem sample were below the limit of detection (<LOD). Conversely, the results show that the dominance of various heavy metals in leaves of plant followed the sequence: P > Mg > Fe > Mn > Zn > Zn > Cd > Cr > Se > Cu > As. Elements such as Co, Mo, Ni and V in leaves of the plant were below the detection limit (<LOD).

**Figure 3:**
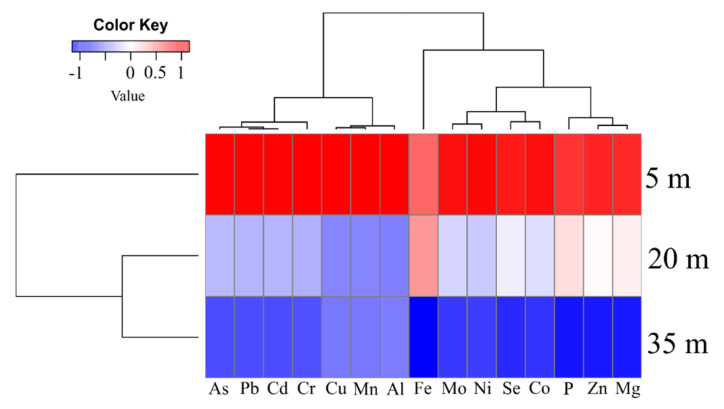
Hierarchical clustering of the means of each metal(loid)s of the soil samples at distances of 5, 20 and 35 m.

At the three soils sampling sites far from the highway, a total of 15 elements were quantified. The concentrations of elements in the soil presented in greater proportions in the distance of 5m in relation to 20 and 35 m (Figure 3). For a distance of 5 m, the concentrations of the elements in the soil decreased in the order of Mg > Fe > Zn > Cu > Mn > Al > Ni > Mo > Cr > Cd > As > P > Co > Se > Pb. For the distance of 20 m, they were: Mg > Fe > Zn > Cu > Al > Mn > Ni > Mo > Cr > P > Cd > As > Co > Se > Pb (Figure 3). For a distance of 35 m, the elements in the ground decreased in Mg > Fe > Zn > Cu > Al > Mn > Ni > Mo > Cu > P > As > Cd > Se > Co > Pb (Figure 3).

**Figure 4:**
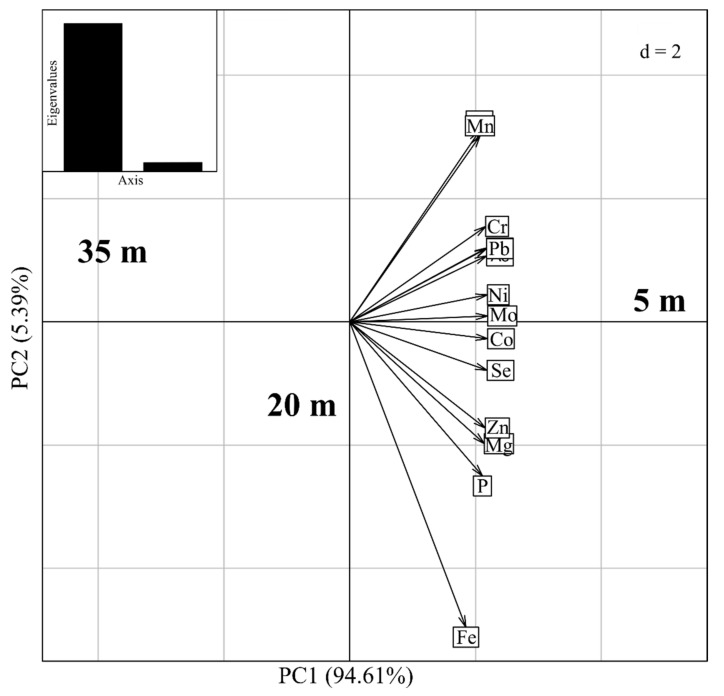
Principal component analysis (PCA) of each metal(loid) of the soil samples at distances of 5, 20 and 35 m.

When the concentrations of elements in the soil were evaluated in relation to different distances of (5, 20 and 35 m) by the Principal Component Analysis (PCA), we found that the elements presented higher trends and proportions at the distance of 5 m with 94.61% on the axis 1 and 5.39% on axis 2 (Figure 4).

### 3.2. Transfer Factor

The transfer factor (TF) of the metal(loid) concentrations in the soils for the tree trunk xylem sap is presented in Table 3. The concentration of elements in the trunk xylem sap in units of mg/L were converted to mg/kg according to Ref. [45]. Here, the three distances of the sampling sites of soils (D1, D2 and D3) were considered in the calculation of the transfer factor. In the stem xylem sap of *D. alata*, elements such as, Cd, Co, Cr. Mo, Ni and Pb are not quantified, therefore, the transfer factor of chemical elements in soils collected at three distances from the sampling sites (D1, D2 and D3) to the trunk xylem sap are not calculated. In addition, the transfer of chemical elements from soils collected at three distances from the sampling sites to leaves plants is shown in Table 4. The elements Co, Mo, Ni and Pb are not quantified in the leaves of *D. alata*; therefore, the calculation of the chemical element transfer factor in soils was not performed (Table 4).

According to Table 3, for the distances of the sampling site of 5 m (D1) from highway edge, the values of soil-to-xylem sap TF decreased in the following order: P (12.97) > Mg (1.48) > Se (0.58) > Mn (0.44) > Al (0.28) > Fe (0.35). For a distance of 5 m, the transfer factors of Mg and P from soil to the trunk xylem sap were greater than 1. Conversely, for the distances of 20 m (D2) and 35 m (D3) from highway edge, the values of transfer factors decreased in the order: P (14.35) > Mg (1.54) > Mn (1.15) > Se (0.64) > Al (0.39) > Fe (0.04), and P (17.17) > Mg (1.63) > Mn (1.23) > Se (0.680) > Al (0.40) > Fe (0.04). For the distances of 20 and 35 m, only the transfer factors of P, Mg and Mn from soil to the sap were greater than 1. All other cases, the TFs were below 1.

Variations in the values of the soil–leaf transfer factor with increasing distance from the highway were studied at distances of 5, 20 and 35 m from highway edge (see Figure 1 and Section 2.1 and Section 2.2). In Table 4, the results revealed that the soil–leaf transfer factor decreased in the following order:

- For distances of 5 m from highway edge: P (1.99) > Mg (0.14) > Al (0.05) > Mn (0.04) > Fe (0.03) > Se (0.02) > Cd (0.02) > Cr (0.01). The TF value for P is greater than 1.
- For distances of 20 m from highway edge: P (2.21) > Mg (0.14) > Mn (0.12) > Al (0.07) > Fe (0.03) > Cd (0.02) > Se (0.02) > Cr (0.01). For P, *TF* > 1.
- For distances of 35 m from highway edge: P (2.64) > Mg (0.15) > Mn (0.13) > Al (0.07) > Fe (0.04) > Cd (0.03) > Se (0.02) > Cr (0.02). Only for Al, the value of the *TF* > 1. For this distance, with excess of Al, all *TF* values < 1.

### 3.3. Health Risk Assessment

Estimated daily intake (EDI) for an adult person (female and male) with age of 50 years and 70 kg body weight, as well as EDI for adolescents (female and male) with 12 years and 40 kg body weight, both compared to the upper level of tolerable intake (UL) are presented in Table 5.

Estimated daily intake (EDI) of heavy metals through trunk xylem sap consumption by adults followed the order Mg > P > Mn > Al > Se > Fe > Zn > Cu (Table 5), while for adolescents was in the order Mg > P > Mn > Al > Se > Fe > Zn > Cu. The UL for aluminum in adults and adolescents has not been established by DRI. However, the daily consumption of Cu, Fe, Mg, Mn, P, Se and Zn in trunk xylem sap by adults and adolescents in Table 5 was below the ULs values. Thus, this does not represent a risk of adverse health effects for adults and adolescents.

The non-carcinogenic risks from consumption of trunk xylem sap for adults and adolescents were assessed based on the HQs. The HQs are presented in Table 6. The HQ values of Al, Cu, Fe, Mg and Zn for adults did not exceed 1; however, HQ values of Mn, P and Se were greater than 1. Conversely, HQ values of P due to consumption of xylem sap by children was greater than 1, and HQ < 1 for the elements Al, Cu, Fe, Mg, Mn, Se and Zn.

## 4. Discussion

The concentration of elements as Mg, Fe, Zn, Cu, Mn, Al, Ni, Mo, Cr, Cd, As, P, Co, Se and Pb in the soil collection sites near at 5 m from highway is higher than 20 and 35 m of distances from highway (Table 1) (See Figure 3 and Figure 4). Through cluster analysis, we observed that the sampling site closest to the 5 m road had higher proportions of metal(loid)s in the soil samples and with PCA it also indicated that the closer to the road, the greater the contaminant exposures potentially toxic. Our results corroborate the observations of Swaileh et al. [46], where high concentrations of Cu, Cd, Pb and Zn were determined in roadside soil, plant and land snail samples along Nablus-Ramallah main road in the West Bank. In addition, according to the results of concentrations of zinc, lead and cadmium in deposits, roadside soil and autochthonous plants (Graminaceae) gathered at the vicinity of a highway in France, the highway induces a contamination on the surrounding environment, up to 320 m, but with the maximum contamination observed between 5 and 20 m [47]. The levels of chemical elements measured in our study at 5, 20 and 35 m at soil collection sites away from the highway (see Figure 3 and Figure 4), and those published in Ref. [47] for distances of 5, 20 and 320 m decrease with increasing distance from the highway. Furthermore, the results presented in Figure 3 are in agreement with those published in Ref. [10] on plants, that is, eleven chemical elements were quantified in the pulp of the fruits of the *C. adamantium* plant collected at two different locations on the road, in which they also observed that the behavior of the concentration of chemical elements (K, P, As, Se, Fe, Mo, Zn, Co, Ni, Mn and Cr) decreased from the roadside (500 m) to the bush (1000 m) (See Figure 2 in Ref. [10]).

The highway can affect plants over long distances. According to study of Kuklová et al. [48], there are effects of expressways located at a distance of 30 to 8100 m on physiology and risk elements content in plants (*Quercus cerris* L., *Prunus spinosa* L., *Melica uniflora* Retz.) and soils. The effect of distance from the road on the content of cadmium (Cd) in soils indicated an increase of the element in mineral layers in the 30 m variant; however, excessive Cd values were recorded in O-horizons and in the background zone. Such results in Ref. [48] also corroborate those obtained in our study (Table 1), that is, at 30, there is a presence of metals due to the traffic of vehicles on the highway.

The contamination of environment with heavy metals emitted from automobiles due to brake linings, tires, as well as road pavement and exhaust fumes have been highlighted in other studies [25,49]. According to Carrero et al. [50], the concentration of Cd, Cr, Cu, Mo, Ni, Pb, Sb, Sn and Zn in soil have different pollution sources and reach roadside soils by road run off. The soil samples studied in our research was carried out near the landfill and bean plantation, thus, soils and plants can be contaminated. According to our results, despite the concentration of Cr, Mn and Pb being lower than those reported Conama/Brazil, China and USA, the contents of Al, As, Cd, Co, Fe, Mg, Mo, Ni, Cr, Se, Mn and P are considerably higher than those reported by these countries.

The leaves in comparison with xylem sap, exhibited lesser concentration of Al, Cu, Mg, Mn, P, Se and Zn. However, elements such as, Cd and Cr were quantified only in leaves. Thus, the results show that the *D. alata* plant has a tendency to accumulate As, Cd and Cr in its leaf tissues. Other plant species accumulate heavy metals in their leaves; a study revealed that for cassava leaves, median values for Zn (107.1 mg/kg), Cu (131 mg/kg), Co (5.2 mg/kg) and As (0.58 mg/kg) in contaminated area are significantly higher compared with cassava leaves in the uncontaminated area (Zn: 50.4; Cu: 20; Co: 1.1; As: 0.23, all in mg/kg). In addition, the arsenic and copper contents in cassava leaves are high and even higher than the contents of this element in the peeled tuber [51].

In our study, the concentrations of Al, Cu, Fe, Mg, Mn, P, Se and Zn in the xylem sap are much higher than the concentration of this same element in the leaves. The Al and Cu concentrations in the sap of the plant trunk were 5.6 and 8.5 times, respectively, above than those quantified in the plant leaves, while the Fe contents in the trunk xylem sap (6.6 ± 0.30 mg/L) are approximately equal to the leaves (6.50 ± 0.52 mg/L). The evaluation of the data showed that the level of Mg exceeded approximately 10.7 times above of the leaves, and the concentration of Mn in trunk xylem sap was 9.5 times highest than leaves. In addition, results in Table 2 show that in trunk xylem sap, the P, Se and Zn levels were higher by 6.5, 29.6 and 3.18 times, respectively, above the concentrations in leaves.

Numerous processes in the xylem sap and leaves of the of the medicinal plant *Dipteryx alata*, which are not subject of this manuscript, can influence heavy metal transport and redistribution processes on the whole plant level [52]. Overall, the concentration of chemical elements in the xylem varies and depends on the pH of the soil [53], sampling period [54], sampled organ, the availability of soil nutrients [55], and the soil water potential [54]. According to studies, heavy metals can be mobilized in the xylem sap through the combination of the fraction stored in roots and old tissues with that recently absorbed by roots from soil [56,57].

The concentration of metals such as Fe, Ni, Mn, Zn, Cd and Cu was found in several studies involving different species of plants. In fact, concentrations of chemical elements as Fe, Se, Zn, Cu, Mn, Cd, Cu, Pb, As, Al and Ni found in the xylem sap, phloem sap and leaf apoplastic fluid of different plant species are different between them [52,58]. However, our results on xylem sap are according to results published by Dambrine et al. [54], in which higher concentration of Mg and P are present in xylem sap.

The transfer factor (TF) is inversely proportional to the value of chemical elements quantified in the soil (Equation (1)). As shown in Figure 3 and Figure 4, the greater the distance between the soil collection sites and the highway, the smaller the concentration of chemical elements in the soil. Therefore, the transfer factor of the concentration of chemical elements from the soil to the sap and flowers for the distance of 35 m are greater than for other distances such as 20 and 5 m. The values of transfer factor of P, Mg and Mn from soil to the xylem sap (Table 3) and transfer factor of P from soil to leaf were (Table 4) greater than 1. Thus, there are higher absorption of P, Mg and Mn from soil by the plant and higher suitability of the plant for phytoremediation and phytoextraction. All other cases, the TFs were below 1, indicating this of the plant species had not a phytoremediation potential, that is, lower values indicate poor response of plants toward metal absorption and the plant could be used for human consumption [59]. The difference in TF values between locations may be related to soil mineral management, presence of heavy metals and soil properties.

The P, Mg and Mn accumulation capacities in trunk xylem sap and leaf of the plant indicated that *D. alata* can be used as possible bioindicators of P, Mg and Mn pollution. The high P, Mg and Mn content in the trunk xylem sap and leaf of the plant is in agreement with the soils near the around the highway, the main polluted area, being highly polluted with P, Mg and Mn. High concentrations of phosphorus are toxic to plants [60] and animal [61]. In addition, excess Mn and Mg can be toxic to plants and humans [62,63].

The mobility of Cd, Cr, Cu, Pb, Ni, Fe, and Zn in the soil from anthropogenic sources in landfill leachate and soils around landfill environments is widely reported and is associated with the migration of landfill leachate [64,65,66,67]. In addition, the mobility and bioavailability of Pb, Cd, Zn, and Cu and their geochemical forms widely varied according to pH, soil organic matter, biochar types, and application rates [66]. According to Awad et al. [68], the risk of heavy metals increases in contaminated soil with the increase in the soluble form. Thus, it became necessary to monitor the different changes of the metal forms. Therefore, the presence of these metals in the soil found in Table 1 may come from landfills, pesticides, fuel combustion residues and petrochemicals [22,67,68,69].

In addition, in 2018, according to Environmental protection agency (EPA) based on aluminum association industry statistics to estimate aluminum packaging generation; landfills received approximately 2.7 million tons of aluminum [70]. High values of element Mg, Fe, Zn, Cu, Mn, Al, Ni, Mo, Cr, Cd, As, P, Co, Se and Pb in soil samples at 5 m (20 and 35 m) from the highway can be explained due to fuels, lubricating oils, fertilizers, tires and vehicle brake disc [22,23,24,25,26].

In the present study, the daily average consumption of sap 250 mg/day for adults and 50 mg/day for adolescents for 90 days/year may pose risks and hazards to humans. The *HQ* values of Mn, P and Se due to trunk xylem sap consumption by adults and P for adolescents are both higher 1, it represents that, in the exposed population, there may be a chronic health risk.

The exposure values of adult’s merit special attention because they account for both higher consumption values and body weight compared with adolescents. However, adolescents are more susceptible to the toxic effects of the heavy metals [71]. Table 6 lists exposure values for some *HQ* calculated. The presences of Al, Cu, Fe, Mg, Mn, P, Se and Zn in xylem sap were the main heavy metals that can cause chronic health risk for both adults and adolescents. Based on the mean HI value (HI > 1), all adolescents and adults were at risk, mainly through ingestion of xylem sap. Thus, the average daily consumption of xylem sap should be less than 250 mg/day for adults and 50 mg/day for adolescents.

The use of the xylem sap of the *D. alata* as tonic should be avoided for children and the elderly, future experimental studies using an animal model of toxicity should be carried out. More rigorous inspection and analysis must be established by several countries, as the trunk xylem sap of some plants can be toxic to humans, although it can be a good indicator of pollution.

The plant has medicinal and nutritional application as explained in the introduction of our article; however, this is the first study that presents the results of the concentration of metal(loid)s in its trunk xylem sap. Research in other countries and in several Brazilian states should be carried out with the trunk xylem sap of the *D. alata* plant in places far from highways and landfills.

If the concentration is high enough, metal(loid)s can accumulate in plants. Then, if contaminated plant or plant parts are consumed by humans, it will be a threat to human health. Studies involving the quantification of heavy metals and metalloids in the soil, analysis of metals in the stalks, leaves and grains of beans, as well as the analysis of sediments and river waters near the sanitary landfills are being carried out. The results obtained in this article will serve as reference values for Brazilian environmental monitoring bodies and possibly for other countries, since they need to monitor these contaminants to assess the impacts of human activities on soil contamination and population health.

The efficiency of ornamental plants for phytoextraction of heavy metals from contaminated soils corrected with organic materials should be encouraged and applied in several countries [72,73]. According to Awad et al., the accumulation of heavy metals by ornamental plants from contaminated agriculture soils is a unique technique that can efficiently reduce the metal load in the food chain [73].

## 5. Conclusions

The concentrations of Mg, Zn, Cu, Mn, Ni, Mo, Cr, Cd, As, P, Co, Se and Pb in the soil near 5 m was higher than 20 and 35 m of distances from the highway. The contents of Al, As, Cd, Co, Fe, Mg, Mo, Ni, Cr, Se, Mn and P in our soils near the landfill and bean plantation were considerably higher than those reported by China and USA.

The concentrations of Al, Cr, Cu, Fe, Mg, Mn, P, Se and Zn in the xylem sap were much higher than the concentration of this same element in the leaves. Thus, the *D. alata* plant has a tendency to accumulate As, Cd and Cr in its leaf tissues.

With the exception of the TF > 1 for P in from soil to leaf, TF for P, Mg and Mn from soil to the xylem sap was below 1, indicating that the plant species had not a phytoremediation potential as the known hyper accumulator plants. The Al, Cu, Fe, Mg, Mn, P, Se and Zn accumulation capacities in trunk xylem sap and leaf of the plant indicated that *D. alata* can be used as possible bioindicators of pollution.

The high values of hazard quotient (*HQ* > 1) recorded in this study represented that in the exposed population there may be a chronic health risk when ingesting the sap of this plant for 90 days/year. Considering that HI is > 1, the cumulative impact of trunk xylem sap consumption is not ignored.

The safety and efficacy of medicinal plant must be proven by clinical data. Future studies with animal models should be carried out, as well as toxicity tests involving the sap from the trunk of the *D. alata* plant.

## Figures and Tables

**Table 1:** Concentrations of elements in soil (median and interquartile deviation) compared to Conama/Brazil [43], soil in China and USA [44].

Element	Concentrations of Elements in Soil: (Distances from the Sampling Sites to Highway Edge) (mg/kg·dw)			Conama/Brazil (mg/kg)	China (mg/kg)	USA (mg/kg)
	5 m	20 m	35 m			
Al	79.56|6.35 a	54.65|1.87 a	54.44|1.50 a	*	6.4	4.7
As	20.85|0.71 a	15.84|0.75 b	14.10|0.94 b	15	9.2	5.2
Cd	24.26|1.16 a	16.06|0.91 b	12.88|0.88 b	1.3	0.07	***
Co	15.12|0.20 a	13.27|0.22 b	12.09|0.12 b	35	11	6.7
Cr	28.47|0.46 a	20.28|0.44 b	18.11|0.18 b	*	54	37
Cu	160.33|0.88 a	73.16|0.48 b	70.65|0.56 b	200	20	17
Fe	188.00|0.88 a	183.76|6.03 a	152.30|0.27 a	*	2.8	1.8
Mg	230.01|0.50 a	220.81|0.51 b	209.40|0.17 b	*	0.67	0.44
Mn	118.10|0.13 a	45.17|0.27 b	42.24|0.24 b	*	432	333
Mo	45.42|0.28 a	28.16|0.56 b	18.64|1.35 b	30	1.2	0.59
Ni	49.86|0.54 a	40.22|0.29 b	35.64|0.50 b	30	23	13
P	20.57|0.58 a	18.60|0.58 b	15.54|0.70 b	*	**	0.02
Se	14.10|0.87 a	13.00|0.02 b	12.14|0.18 b	*	0.22	0.26
Zn	178.85|1.27 a	165.40|0.12 b	150.28|0.07 b	300	67	48
Pb	12.44|0.51 a	7.08|1.33 b	5.64|0.45 b	72	24	16

“a” and “b” mean statistically significant difference; * Values not determined by Conama/Brazil; ** Values not determined by China; *** Values not determined by USA.

**Table 2:** Concentrations of elements in trunk xylem sap and leaves of *D. alata*.

Elements	Trunk Xylem Sap (mg/L)	Leaves (mg/L)
Al	21.40 ± 0.20	3.86 ± 0.39
As	<LOD	0.06 ± 0.07
Cd	<LOD	0.42 ± 0.10
Co	<LOD	<LOD
Cr	<LOD	0.34 ± 0.16
Cu	0.60 ± 0.01	0.07 ± 0.05
Fe	6.60 ± 0.30	6.50 ± 0.52
Mg	341.00 ± 2.40	31.89 ± 1.19
Mn	52.20 ± 0.50	5.51 ± 0.24
Mo	<LOD	<LOD
Ni	<LOD	<LOD
P	266.90 ± 0.40	41.07 ± 0.16
Se	8.30 ± 0.40	0.29 ± 0.14
V	<LOD	<LOD
Zn	1.40 ± 0.10	0.44 ± 0.03

<LOD—analyte concentrations were below the limits of detection.

**Table 3:** Transfer factor of chemical elements in soils collected at three distances from the sampling sites from highway edge (D1, D2 and D3) to the trunk xylem sap.

Element	Transfer Factor of Concentrations of Elements in Soil to Trunk Xylem Sap of the Plant		
	Distances from the Sampling Sites to Highway Edge		
	5 m	20 m	35 m
Al	0.28	0.39	0.40
Cu	0.00	0.00	0.00
Fe	0.03	0.04	0.04
Mg	1.48	1.54	1.63
Mn	0.44	1.15	1.23
P	12.97	14.35	17.17
Se	0.58	0.64	0.68
Zn	0.00	0.00	0.00

**Table 4:** Transfer factor of chemical elements in soils collected at three distances from the sampling sites from highway edge (D1, D2 and D3) to plant leaves.

Element	Transfer Factor of Concentrations of Elements in Soil to Leaves of the Plant		
	Distances from the Sampling Sites to Highway Edge		
	5 m	20 m	35 m
Al	0.05	0.07	0.07
As	0.00	0.00	0.00
Cd	0.02	0.02	0.03
Cr	0.01	0.01	0.02
Cu	0.00	0.00	0.00
Fe	0.03	0.03	0.04
Mg	0.14	0.14	0.15
Mn	0.04	0.12	0.13
P	1.99	2.20	2.64
Se	0.02	0.02	0.02
Zn	0.00	0.00	0.00

**Table 5:** Estimated daily intake (*EDI*) of each element via consumption of trunk xylem sap of the tree compared to Upper level of tolerable intake (UL) [35].

Elements	Adults (Female/Male) 50 Years (70 kg)		Adolescents (Female/Male) 12 Years (40 kg)	
	Estimated Daily Intake (mg/kg/day)	Upper Level of Tolerable Intake (UL) (mg/day)	Estimated Daily Intake (mg/kg/day)	Upper Level of Tolerable Intake (UL) (mg/day)
Al	0.07 ± 7.1 × 10^−4^	ND	0.03 ± 2.5 × 10^−4^	ND
Cu	2.14 × 10^−3^ ± 3.5 × 10^−5^	10	7.5 × 10^−4^ ± 1.0 × 10^−5^	5
Fe	0.02 ± 0.011	45	8.25 × 10^−3^ ± 0.04	40
Mg	1.28 ± 8.57 × 10^−3^	350	0.43 ± 3.0 × 10^−3^	350
Mn	0.18 ± 1.78 × 10^−3^	11	0.06 ± 6.25 × 10^−4^	6
P	0.95 ± 1.42 × 10^−3^	4000	0.3 ± 5.0 × 10^−4^	4000
Se	0.03 ± 1.42 × 10^−3^	0.4	0.01 ± 5.0 × 10^−4^	0.28
Zn	5.0 × 10^−3^ ± 3.5 × 10^−4^	40	1.75 × 10^−3^ ± 1.25 × 10^−4^	23

**Table 6:** Target hazard quotient (*HQ*) due to consumption of trunk xylem sap of the tree by adults and children and chronic hazard index (HI).

Elements	Adults 50 Years (70 kg)	Children 12 Years (40 kg)
	*HQ*	*HQ*
Al	1.88 × 10^−2^ ± 1.80 × 10^−4^	6.59 × 10^−3^ ± 6.16 × 10^−5^
Cu	0.01 ± 2.15 × 10^−4^	4.4 × 10^−3^ ± 7.3 × 10^−5^
Fe	8.27 × 10^−3^ ± 3.76 × 10^−3^	2.9 × 10^−3^ ± 1.3 × 10^−3^
Mg	0.03 ± 1.92 × 10^−4^	9.55 × 10^−3^ ± 6.72 × 10^−5^
Mn	1.91 ± 0.0182	0.68 ± 6.42 × 10^−3^
P	11,751.90 ± 17.50	4105.47 ± 6.16
Se	1.43 ± 0.07	0.507 ± 0.024
Zn	4.10 × 10^−3^ ± 2.93 × 10^−4^	1.43 × 10^−3^ ± 1.02 × 10^−4^
**Chronic hazard index (HI)**	11,755.31 ± 17.59	4106.60 ± 6.19

## Data Availability

The data used to support the findings of this study are available from the corresponding author upon request.
